# 5-Meth­oxy-1,2′,3-trimethyl-4,6-dioxa-2-aza­spiro­[bicyclo­[3.2.0]hept-2-ene-7,4′-isoquinoline]-1′,3′(2′*H*,4′*H*)-dione

**DOI:** 10.1107/S1600536811016266

**Published:** 2011-05-07

**Authors:** Hoong-Kun Fun, Ching Kheng Quah, Chengmei Huang, Haitao Yu

**Affiliations:** a X-ray Crystallography Unit, School of Physics, Universiti Sains Malaysia, 11800 USM, Penang, Malaysia; b School of Chemistry and Chemical Engineering, Nanjing University, Nanjing 210093, People’s Republic of China

## Abstract

In the isoquinoline ring system of the title mol­ecule, C_16_H_16_N_2_O_5_, the *N*-heterocyclic ring is in a half-boat conformation. The dioxaaza­spiro ring is essentially planar [maximum deviation = 0.022 (1) Å] and forms a dihedral angle of 24.56 (4)° with the benzene ring.

## Related literature

For general background to and the potential biological activity of the title compound, see: Du *et al.* (2008 ▶); Chen *et al.* (2006 ▶); Yu *et al.* (2010 ▶); Harris *et al.* (2005 ▶); Zhang *et al.* (2004 ▶); Wang *et al.* (2010 ▶); Huang *et al.* (2011 ▶). For the stability of the temperature controller used in the data collection, see: Cosier & Glazer (1986 ▶). For standard bond-length data, see: Allen *et al.* (1987 ▶). For ring conformations, see: Cremer & Pople (1975 ▶). For related structures, see: Fun *et al.* (2011*a* ▶,*b* ▶,*c* ▶).
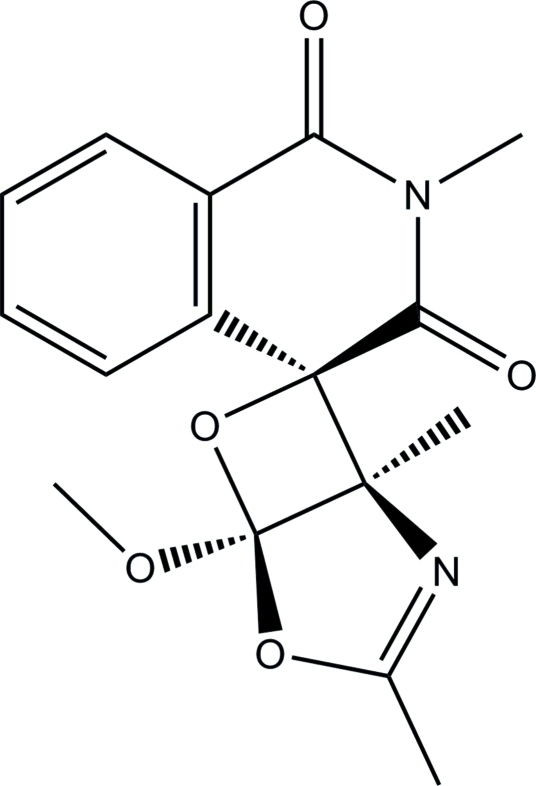


## Experimental

### 

#### Crystal data

C_16_H_16_N_2_O_5_
*M* *_r_* = 316.31
Monoclinic, 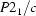

*a* = 7.3643 (1) Å
*b* = 29.8703 (6) Å
*c* = 6.7802 (1) Å
β = 105.294 (1)°
*V* = 1438.65 (4) Å^3^
*Z* = 4
Mo *K*α radiation
μ = 0.11 mm^−1^
*T* = 100 K
0.46 × 0.31 × 0.27 mm

#### Data collection

Bruker SMART APEXII CCD area-detector diffractometer
Absorption correction: multi-scan (*SADABS*; Bruker, 2009 ▶) *T*_min_ = 0.951, *T*_max_ = 0.971
44501 measured reflections
8265 independent reflections
6842 reflections with *I* > 2σ(*I*)
*R*_int_ = 0.033

#### Refinement

*R*[*F* ^2^ > 2σ(*F* ^2^)] = 0.049
*wR*(*F* ^2^) = 0.126
*S* = 1.08
8265 reflections
212 parameters
H-atom parameters constrained
Δρ_max_ = 0.50 e Å^−3^
Δρ_min_ = −0.37 e Å^−3^

### 

Data collection: *APEX2* (Bruker, 2009 ▶); cell refinement: *SAINT* (Bruker, 2009 ▶); data reduction: *SAINT*; program(s) used to solve structure: *SHELXTL* (Sheldrick, 2008 ▶); program(s) used to refine structure: *SHELXTL*; molecular graphics: *SHELXTL*; software used to prepare material for publication: *SHELXTL* and *PLATON* (Spek, 2009 ▶).

## Supplementary Material

Crystal structure: contains datablocks global, I. DOI: 10.1107/S1600536811016266/lh5241sup1.cif

Structure factors: contains datablocks I. DOI: 10.1107/S1600536811016266/lh5241Isup2.hkl

Supplementary material file. DOI: 10.1107/S1600536811016266/lh5241Isup3.cml

Additional supplementary materials:  crystallographic information; 3D view; checkCIF report

## Figures and Tables

**Fig. 1.**
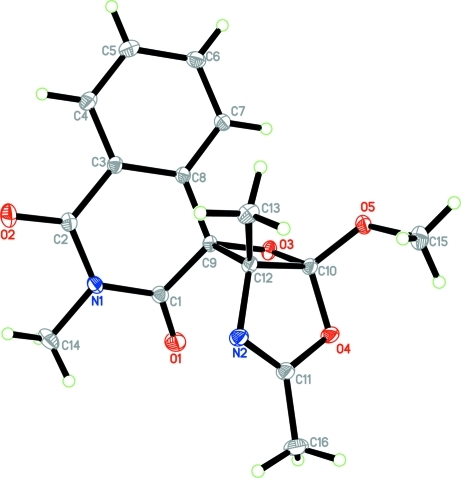
The molecular structure of the title compound showing 50% probability displacement ellipsoids for non-H atoms.

## supplementary crystallographic information

### Comment

Isoquinoline-1,3,4-trione derivatives have been reported to be a type of small molecular inhibitor against caspase-3 which can promote apoptosis of the cells (Du *et al.*, 2008; Chen *et al.*, 2006). Photoreactions of isoquinoline-1,3,4-trione could lead to structurally important motifs (Yu *et al.*, 2010). Oxazole ring is found in some bioactive natural products such as Annuloline and Ostreogrycin A. Oxazoles can be used to inhibit the activity of malignant tumors (Harris *et al.*, 2005). Since a lot of natural products especially the alkaloids containing isoquinoline or oxazole ring are bioactive, convenient method to construct such moieties is of current research interest (Zhang *et al.*, 2004; Wang *et al.*, 2010). The title compound which was derived from isoquinoline-1,3,4-trione and oxazoles (Huang *et al.*, 2011) may has a potential use in biochemical and pharmaceutical fields. We report in this paper the crystal structure of the title compound with a relative configuration of (1S^*^, 4'S^*^, 5R^*^).

In the title racemic compound, Fig. 1, atoms C9, C10 and C12 are the stereo centers. The isoquinoline ring system (N1/C1-C9) is not completely planar, the *N*-heterocyclic ring (N1/C1-C3/C8/C9) being distorted towards a half-boat conformation with atom C9 deviating by 0.231 (1) Å from the mean plane through the remaining atoms, puckering parameters (Cremer & Pople, 1975) Q = 0.3501 (8) Å, Θ =  112.83 (13)° and φ = 282.17 (13)°. The dioxa-2-azaspiro ring (N2/O4/C10-C12) is essentially planar [maximum deviation of 0.022 (1) Å at atom C10] and it inclines at a dihedral angle of 24.56 (4)° with the benzene ring (C3-C8). Bond lengths (Allen *et al.*, 1987) and angles are within normal ranges and comparable to related structures (Fun *et al.*, 2011*a*,*b*,*c*). In the crystal of the title compound, unlike in our previously determined structures mentioned above, there are no significant intermolecular hydrogen bonds.

### Experimental

The title compound was the main product from the photoreaction between isoquinoline-1,3,4-trione and 4-methyl-5-methoxy-2-methyloxazole. The compound was purified by flash column chromatography with ethyl acetate/petroleum ether (1:3) as eluents. X-ray quality crystals of the title compound was obtained from slow evaporation of an acetone and petroleum ether solution (1:4) (*m.p.* 437-439 K).

### Refinement

All H atoms were positioned geometrically and refined using a riding model with C–H = 0.93 - 0.96 Å and *U*_iso_(H) = 1.2 or 1.5 *U*_eq_(C). A rotating-group model was applied for the methyl groups. The highest residual electron density peak is located at 0.77 Å from C9 and the deepest hole is located at 0.52 Å from C2.

### Crystal data

**Table:**

C_16_H_16_N_2_O_5_	*F*(000) = 664
*M**_r_* = 316.31	*D*_x_ = 1.460 Mg m^−^^3^
Monoclinic, *P*2_1_/*c*	Mo *K*α radiation, λ = 0.71073 Å
Hall symbol: -P 2ybc	Cell parameters from 9541 reflections
*a* = 7.3643 (1) Å	θ = 2.7–38.9°
*b* = 29.8703 (6) Å	µ = 0.11 mm^−^^1^
*c* = 6.7802 (1) Å	*T* = 100 K
β = 105.294 (1)°	Block, colourless
*V* = 1438.65 (4) Å^3^	0.46 × 0.31 × 0.27 mm
*Z* = 4	

### Data collection

**Table:**

Bruker SMART APEXII CCD area-detector diffractometer	8265 independent reflections
Radiation source: fine-focus sealed tube	6842 reflections with *I* > 2σ(*I*)
graphite	*R*_int_ = 0.033
φ and ω scans	θ_max_ = 39.1°, θ_min_ = 2.7°
Absorption correction: multi-scan (*SADABS*; Bruker, 2009)	*h* = −13→11
*T*_min_ = 0.951, *T*_max_ = 0.971	*k* = −52→52
44501 measured reflections	*l* = −10→12

### Refinement

**Table:**

Refinement on *F*^2^	Primary atom site location: structure-invariant direct methods
Least-squares matrix: full	Secondary atom site location: difference Fourier map
*R*[*F*^2^ > 2σ(*F*^2^)] = 0.049	Hydrogen site location: inferred from neighbouring sites
*wR*(*F*^2^) = 0.126	H-atom parameters constrained
*S* = 1.08	*w* = 1/[σ^2^(*F*_o_^2^) + (0.0561*P*)^2^ + 0.4048*P*] where *P* = (*F*_o_^2^ + 2*F*_c_^2^)/3
8265 reflections	(Δ/σ)_max_ = 0.001
212 parameters	Δρ_max_ = 0.50 e Å^−^^3^
0 restraints	Δρ_min_ = −0.37 e Å^−^^3^

### Special details

**Table:**

Experimental. The crystal was placed in the cold stream of an Oxford Cryosystems Cobra open-flow nitrogen cryostat (Cosier & Glazer, 1986) operating at 100.0 (1) K.
Geometry. All esds (except the esd in the dihedral angle between two l.s. planes) are estimated using the full covariance matrix. The cell esds are taken into account individually in the estimation of esds in distances, angles and torsion angles; correlations between esds in cell parameters are only used when they are defined by crystal symmetry. An approximate (isotropic) treatment of cell esds is used for estimating esds involving l.s. planes.
Refinement. Refinement of F^2^ against ALL reflections. The weighted R-factor wR and goodness of fit S are based on F^2^, conventional R-factors R are based on F, with F set to zero for negative F^2^. The threshold expression of F^2^ > 2sigma(F^2^) is used only for calculating R-factors(gt) etc. and is not relevant to the choice of reflections for refinement. R-factors based on F^2^ are statistically about twice as large as those based on F, and R- factors based on ALL data will be even larger.

### Fractional atomic coordinates and isotropic or equivalent isotropic displacement parameters (Å^2^)

**Table:**

	*x*	*y*	*z*	*U*_iso_*/*U*_eq_	
O1	0.98692 (10)	0.07763 (2)	1.21672 (11)	0.01961 (12)	
O2	0.97185 (10)	0.22835 (2)	1.29761 (11)	0.02036 (12)	
O3	0.64272 (8)	0.074342 (18)	0.93351 (9)	0.01245 (10)	
O4	0.80974 (8)	0.037356 (19)	0.72911 (9)	0.01405 (10)	
O5	0.50850 (8)	0.06418 (2)	0.59672 (9)	0.01442 (10)	
N1	0.99366 (9)	0.15373 (2)	1.23860 (10)	0.01288 (11)	
N2	0.98329 (9)	0.10159 (2)	0.78175 (11)	0.01329 (11)	
C1	0.91387 (11)	0.11323 (3)	1.15734 (11)	0.01266 (12)	
C2	0.89437 (11)	0.19420 (3)	1.22005 (11)	0.01288 (12)	
C3	0.68983 (10)	0.19200 (2)	1.11676 (11)	0.01123 (11)	
C4	0.57505 (11)	0.22751 (2)	1.14283 (12)	0.01418 (12)	
H4A	0.6279	0.2526	1.2171	0.017*	
C5	0.38157 (12)	0.22526 (3)	1.05764 (13)	0.01604 (13)	
H5A	0.3048	0.2489	1.0743	0.019*	
C6	0.30314 (11)	0.18742 (3)	0.94723 (12)	0.01532 (13)	
H6A	0.1739	0.1860	0.8893	0.018*	
C7	0.41660 (11)	0.15171 (3)	0.92290 (11)	0.01323 (12)	
H7A	0.3630	0.1264	0.8506	0.016*	
C8	0.61080 (10)	0.15391 (2)	1.00719 (10)	0.01055 (11)	
C9	0.73909 (10)	0.11699 (2)	0.97884 (11)	0.01041 (11)	
C10	0.67607 (10)	0.07134 (2)	0.73762 (11)	0.01101 (11)	
C11	0.98054 (11)	0.05895 (3)	0.76219 (12)	0.01374 (12)	
C12	0.79021 (10)	0.11558 (2)	0.76495 (11)	0.01075 (11)	
C13	0.72971 (12)	0.15463 (3)	0.62183 (12)	0.01482 (13)	
H13A	0.7495	0.1475	0.4909	0.022*	
H13B	0.8025	0.1806	0.6771	0.022*	
H13C	0.5986	0.1607	0.6063	0.022*	
C14	1.18102 (12)	0.15176 (3)	1.38319 (13)	0.01961 (15)	
H14A	1.2585	0.1311	1.3338	0.029*	
H14B	1.1695	0.1420	1.5143	0.029*	
H14C	1.2376	0.1809	1.3961	0.029*	
C15	0.51787 (13)	0.05535 (3)	0.39061 (13)	0.02037 (15)	
H15A	0.3927	0.0530	0.3024	0.031*	
H15B	0.5840	0.0278	0.3874	0.031*	
H15C	0.5831	0.0794	0.3447	0.031*	
C16	1.14282 (12)	0.02931 (3)	0.76728 (17)	0.02177 (17)	
H16A	1.2575	0.0462	0.8101	0.033*	
H16B	1.1314	0.0173	0.6333	0.033*	
H16C	1.1448	0.0053	0.8618	0.033*	

### Atomic displacement parameters (Å^2^)

**Table:**

	*U*^11^	*U*^22^	*U*^33^	*U*^12^	*U*^13^	*U*^23^
O1	0.0194 (3)	0.0141 (2)	0.0220 (3)	0.0030 (2)	−0.0004 (2)	0.0036 (2)
O2	0.0186 (3)	0.0147 (3)	0.0249 (3)	−0.0053 (2)	0.0006 (2)	−0.0044 (2)
O3	0.0150 (2)	0.0102 (2)	0.0132 (2)	−0.00369 (17)	0.00555 (17)	−0.00184 (17)
O4	0.0112 (2)	0.0101 (2)	0.0215 (3)	−0.00064 (17)	0.00536 (18)	−0.00282 (18)
O5	0.0113 (2)	0.0169 (2)	0.0144 (2)	−0.00187 (18)	0.00209 (17)	−0.00453 (19)
N1	0.0106 (2)	0.0138 (3)	0.0126 (2)	−0.00073 (19)	0.00022 (18)	−0.0006 (2)
N2	0.0113 (2)	0.0118 (2)	0.0179 (3)	−0.00080 (19)	0.0059 (2)	−0.0016 (2)
C1	0.0126 (3)	0.0126 (3)	0.0121 (3)	0.0000 (2)	0.0022 (2)	0.0005 (2)
C2	0.0132 (3)	0.0125 (3)	0.0123 (3)	−0.0019 (2)	0.0022 (2)	−0.0002 (2)
C3	0.0124 (3)	0.0104 (3)	0.0106 (2)	−0.0008 (2)	0.0026 (2)	0.0001 (2)
C4	0.0174 (3)	0.0106 (3)	0.0146 (3)	0.0006 (2)	0.0044 (2)	−0.0004 (2)
C5	0.0169 (3)	0.0143 (3)	0.0176 (3)	0.0039 (2)	0.0058 (2)	0.0005 (2)
C6	0.0117 (3)	0.0175 (3)	0.0164 (3)	0.0022 (2)	0.0031 (2)	0.0001 (2)
C7	0.0113 (3)	0.0146 (3)	0.0133 (3)	0.0000 (2)	0.0022 (2)	−0.0016 (2)
C8	0.0114 (3)	0.0107 (3)	0.0095 (2)	−0.0003 (2)	0.00277 (19)	−0.0006 (2)
C9	0.0110 (3)	0.0094 (2)	0.0107 (2)	−0.0013 (2)	0.00248 (19)	−0.0008 (2)
C10	0.0104 (3)	0.0102 (3)	0.0127 (3)	−0.0006 (2)	0.0035 (2)	−0.0015 (2)
C11	0.0116 (3)	0.0123 (3)	0.0183 (3)	−0.0008 (2)	0.0056 (2)	−0.0020 (2)
C12	0.0112 (3)	0.0098 (3)	0.0117 (3)	−0.0006 (2)	0.0040 (2)	−0.0009 (2)
C13	0.0187 (3)	0.0124 (3)	0.0145 (3)	0.0010 (2)	0.0064 (2)	0.0021 (2)
C14	0.0131 (3)	0.0241 (4)	0.0178 (3)	−0.0014 (3)	−0.0026 (2)	0.0012 (3)
C15	0.0215 (4)	0.0237 (4)	0.0149 (3)	−0.0007 (3)	0.0030 (3)	−0.0056 (3)
C16	0.0140 (3)	0.0152 (3)	0.0374 (5)	0.0019 (3)	0.0092 (3)	−0.0041 (3)

### Geometric parameters (Å, °)

**Table:**

O1—C1	1.2116 (10)	C6—C7	1.3914 (11)
O2—C2	1.2183 (9)	C6—H6A	0.9300
O3—C10	1.4158 (9)	C7—C8	1.3946 (10)
O3—C9	1.4512 (9)	C7—H7A	0.9300
O4—C11	1.3784 (9)	C8—C9	1.4972 (10)
O4—C10	1.4257 (9)	C9—C12	1.5921 (10)
O5—C10	1.3636 (9)	C10—C12	1.5508 (10)
O5—C15	1.4416 (10)	C11—C16	1.4804 (11)
N1—C1	1.3933 (10)	C12—C13	1.5075 (10)
N1—C2	1.4012 (10)	C13—H13A	0.9600
N1—C14	1.4679 (10)	C13—H13B	0.9600
N2—C11	1.2799 (10)	C13—H13C	0.9600
N2—C12	1.4574 (10)	C14—H14A	0.9600
C1—C9	1.5206 (10)	C14—H14B	0.9600
C2—C3	1.4857 (10)	C14—H14C	0.9600
C3—C4	1.3961 (10)	C15—H15A	0.9600
C3—C8	1.3990 (10)	C15—H15B	0.9600
C4—C5	1.3907 (12)	C15—H15C	0.9600
C4—H4A	0.9300	C16—H16A	0.9600
C5—C6	1.3941 (12)	C16—H16B	0.9600
C5—H5A	0.9300	C16—H16C	0.9600
			
C10—O3—C9	93.33 (5)	O5—C10—O4	111.53 (6)
C11—O4—C10	105.68 (6)	O3—C10—O4	112.06 (6)
C10—O5—C15	116.23 (6)	O5—C10—C12	125.39 (6)
C1—N1—C2	123.91 (6)	O3—C10—C12	93.25 (5)
C1—N1—C14	116.97 (7)	O4—C10—C12	104.67 (6)
C2—N1—C14	118.08 (7)	N2—C11—O4	118.12 (7)
C11—N2—C12	106.79 (6)	N2—C11—C16	127.02 (7)
O1—C1—N1	121.77 (7)	O4—C11—C16	114.85 (7)
O1—C1—C9	122.53 (7)	N2—C12—C13	112.88 (6)
N1—C1—C9	115.52 (6)	N2—C12—C10	104.58 (6)
O2—C2—N1	120.65 (7)	C13—C12—C10	121.57 (6)
O2—C2—C3	122.79 (7)	N2—C12—C9	113.37 (6)
N1—C2—C3	116.38 (6)	C13—C12—C9	117.72 (6)
C4—C3—C8	120.27 (7)	C10—C12—C9	83.13 (5)
C4—C3—C2	118.63 (7)	C12—C13—H13A	109.5
C8—C3—C2	120.95 (6)	C12—C13—H13B	109.5
C5—C4—C3	119.93 (7)	H13A—C13—H13B	109.5
C5—C4—H4A	120.0	C12—C13—H13C	109.5
C3—C4—H4A	120.0	H13A—C13—H13C	109.5
C4—C5—C6	119.74 (7)	H13B—C13—H13C	109.5
C4—C5—H5A	120.1	N1—C14—H14A	109.5
C6—C5—H5A	120.1	N1—C14—H14B	109.5
C7—C6—C5	120.57 (7)	H14A—C14—H14B	109.5
C7—C6—H6A	119.7	N1—C14—H14C	109.5
C5—C6—H6A	119.7	H14A—C14—H14C	109.5
C6—C7—C8	119.88 (7)	H14B—C14—H14C	109.5
C6—C7—H7A	120.1	O5—C15—H15A	109.5
C8—C7—H7A	120.1	O5—C15—H15B	109.5
C7—C8—C3	119.60 (7)	H15A—C15—H15B	109.5
C7—C8—C9	121.75 (6)	O5—C15—H15C	109.5
C3—C8—C9	118.62 (6)	H15A—C15—H15C	109.5
O3—C9—C8	112.58 (6)	H15B—C15—H15C	109.5
O3—C9—C1	111.58 (6)	C11—C16—H16A	109.5
C8—C9—C1	112.49 (6)	C11—C16—H16B	109.5
O3—C9—C12	90.22 (5)	H16A—C16—H16B	109.5
C8—C9—C12	116.44 (6)	C11—C16—H16C	109.5
C1—C9—C12	111.66 (6)	H16A—C16—H16C	109.5
O5—C10—O3	108.56 (6)	H16B—C16—H16C	109.5
			
C2—N1—C1—O1	−161.40 (8)	O1—C1—C9—C12	−81.94 (9)
C14—N1—C1—O1	6.68 (11)	N1—C1—C9—C12	93.26 (7)
C2—N1—C1—C9	23.35 (10)	C15—O5—C10—O3	−173.45 (6)
C14—N1—C1—C9	−168.56 (7)	C15—O5—C10—O4	−49.51 (9)
C1—N1—C2—O2	178.82 (8)	C15—O5—C10—C12	78.38 (9)
C14—N1—C2—O2	10.86 (11)	C9—O3—C10—O5	−126.97 (6)
C1—N1—C2—C3	3.52 (10)	C9—O3—C10—O4	109.41 (6)
C14—N1—C2—C3	−164.44 (7)	C9—O3—C10—C12	2.15 (6)
O2—C2—C3—C4	−12.66 (11)	C11—O4—C10—O5	142.09 (6)
N1—C2—C3—C4	162.54 (7)	C11—O4—C10—O3	−95.97 (7)
O2—C2—C3—C8	171.75 (7)	C11—O4—C10—C12	3.77 (7)
N1—C2—C3—C8	−13.05 (10)	C12—N2—C11—O4	0.91 (9)
C8—C3—C4—C5	−0.74 (11)	C12—N2—C11—C16	179.69 (8)
C2—C3—C4—C5	−176.36 (7)	C10—O4—C11—N2	−3.21 (9)
C3—C4—C5—C6	0.27 (12)	C10—O4—C11—C16	177.86 (7)
C4—C5—C6—C7	0.53 (12)	C11—N2—C12—C13	−132.59 (7)
C5—C6—C7—C8	−0.85 (12)	C11—N2—C12—C10	1.60 (8)
C6—C7—C8—C3	0.38 (11)	C11—N2—C12—C9	90.33 (7)
C6—C7—C8—C9	−177.87 (7)	O5—C10—C12—N2	−133.99 (7)
C4—C3—C8—C7	0.41 (10)	O3—C10—C12—N2	110.46 (6)
C2—C3—C8—C7	175.93 (7)	O4—C10—C12—N2	−3.35 (7)
C4—C3—C8—C9	178.71 (7)	O5—C10—C12—C13	−4.83 (10)
C2—C3—C8—C9	−5.77 (10)	O3—C10—C12—C13	−120.39 (7)
C10—O3—C9—C8	116.84 (6)	O4—C10—C12—C13	125.81 (7)
C10—O3—C9—C1	−115.57 (6)	O5—C10—C12—C9	113.59 (7)
C10—O3—C9—C12	−2.09 (5)	O3—C10—C12—C9	−1.97 (5)
C7—C8—C9—O3	−23.21 (9)	O4—C10—C12—C9	−115.78 (6)
C3—C8—C9—O3	158.52 (6)	O3—C9—C12—N2	−101.04 (6)
C7—C8—C9—C1	−150.32 (7)	C8—C9—C12—N2	143.45 (6)
C3—C8—C9—C1	31.42 (9)	C1—C9—C12—N2	12.37 (8)
C7—C8—C9—C12	78.99 (8)	O3—C9—C12—C13	124.09 (7)
C3—C8—C9—C12	−99.28 (7)	C8—C9—C12—C13	8.58 (9)
O1—C1—C9—O3	17.35 (10)	C1—C9—C12—C13	−122.51 (7)
N1—C1—C9—O3	−167.44 (6)	O3—C9—C12—C10	1.92 (5)
O1—C1—C9—C8	144.99 (8)	C8—C9—C12—C10	−113.59 (6)
N1—C1—C9—C8	−39.81 (9)	C1—C9—C12—C10	115.32 (6)
